# Aldosterone Modulates the Association between NCC and ENaC

**DOI:** 10.1038/s41598-017-03510-5

**Published:** 2017-06-23

**Authors:** Brandi M. Wynne, Abinash C. Mistry, Otor Al-Khalili, Rickta Mallick, Franziska Theilig, Douglas C. Eaton, Robert S. Hoover

**Affiliations:** 1 Division of Nephrology, Department of Medicine, Emory University, Atlanta, GA 30322 USA; 2 Department of Physiology, Emory University, Atlanta, GA 30322 USA; 3 Department of Medicine, University of Fribourg, Fribourg, Switzerland; 4 Center for Cell and Molecular Signaling, Emory University, Atlanta, GA 30322 USA; 5 Research Service, Atlanta Veteran’s Administration Medical Center, Decatur, GA 30033 USA

## Abstract

Distal sodium transport is a final step in the regulation of blood pressure. As such, understanding how the two main sodium transport proteins, the thiazide-sensitive sodium chloride cotransporter (NCC) and the epithelial sodium channel (ENaC), are regulated is paramount. Both are expressed in the late distal nephron; however, no evidence has suggested that these two sodium transport proteins interact. Recently, we established that these two sodium transport proteins functionally interact in the second part of the distal nephron (DCT2). Given their co-localization within the DCT2, we hypothesized that NCC and ENaC interactions might be modulated by aldosterone (Aldo). Aldo treatment increased NCC and αENaC colocalization (electron microscopy) and interaction (coimmunoprecipitation). Finally, with co-expression of the Aldo-induced protein serum- and glucocorticoid-inducible kinase 1 (SGK1), NCC and αENaC interactions were increased. These data demonstrate that Aldo promotes increased interaction of NCC and ENaC, within the DCT2 revealing a novel method of regulation for distal sodium reabsorption.

## Introduction

Hypertension is a primary cause of death and disability in the United States and worldwide^1^. Understanding the mechanisms that cause hypertension is crucial in combatting this debilitating disease. Increased renal sodium (Na^+^) reabsorption is a primary pathogenic factor. Although the vast majority of Na^+^ is reabsorbed within the proximal tubule, the aldosterone-sensitive distal nephron (ASDN) is where fine-tuning and regulation of Na^+^ handling occurs. The ASDN expresses two primary sodium transport proteins- the thiazide-sensitive sodium-chloride cotransporter (NCC) and the epithelial sodium channel (ENaC). Genetic aberrations in either lead to a disruption in sodium homeostasis and either hyper- or hypotension. Indeed, all currently known inherited and acquired forms of hypertension affect renal sodium (Na^+^) chloride (NaCl) handling, with most involving these two transport proteins^2–4^.

The ASDN is comprised of the late distal convoluted tubule (DCT2), connecting tubule (CNT) and cortical collecting duct (CCD). NCC is expressed fully along the DCT, while ENaC expression begins at the DCT2 and continues throughout the remainder of the ASDN. Studies have demonstrated that NCC and ENaC are co-expressed in one segment of the ASDN, the DCT2^5–12^. However, no studies have suggested any interaction between these two transport proteins. Indeed, anatomical and/or functional interactions between channels and transporters are rare. However, our laboratory has recently demonstrated that NCC and ENaC physically associate *in vivo* and *in vitro* using both co-immunoprecipitation (CoIP) and electron microscopy (EM) immunogold labeling. Utilizing a mammalian two-hybrid system we then demonstrated a direct interaction between these proteins^12^. Additionally, inhibition of NCC function with thiazides reduced ENaC open probability, confirming a functional interaction of these proteins^12^. However, mechanisms that might regulate this interaction are unknown. Given their location in the ASDN, we hypothesized that aldosterone might modulate their interactions. Here we report that this hypothesis is true; the interaction of these two key Na^+^ transport proteins is regulated by aldosterone.

## Results

### EM demonstrates increased NCC and ENaC interaction after aldosterone stimulation

**Figure 1:**
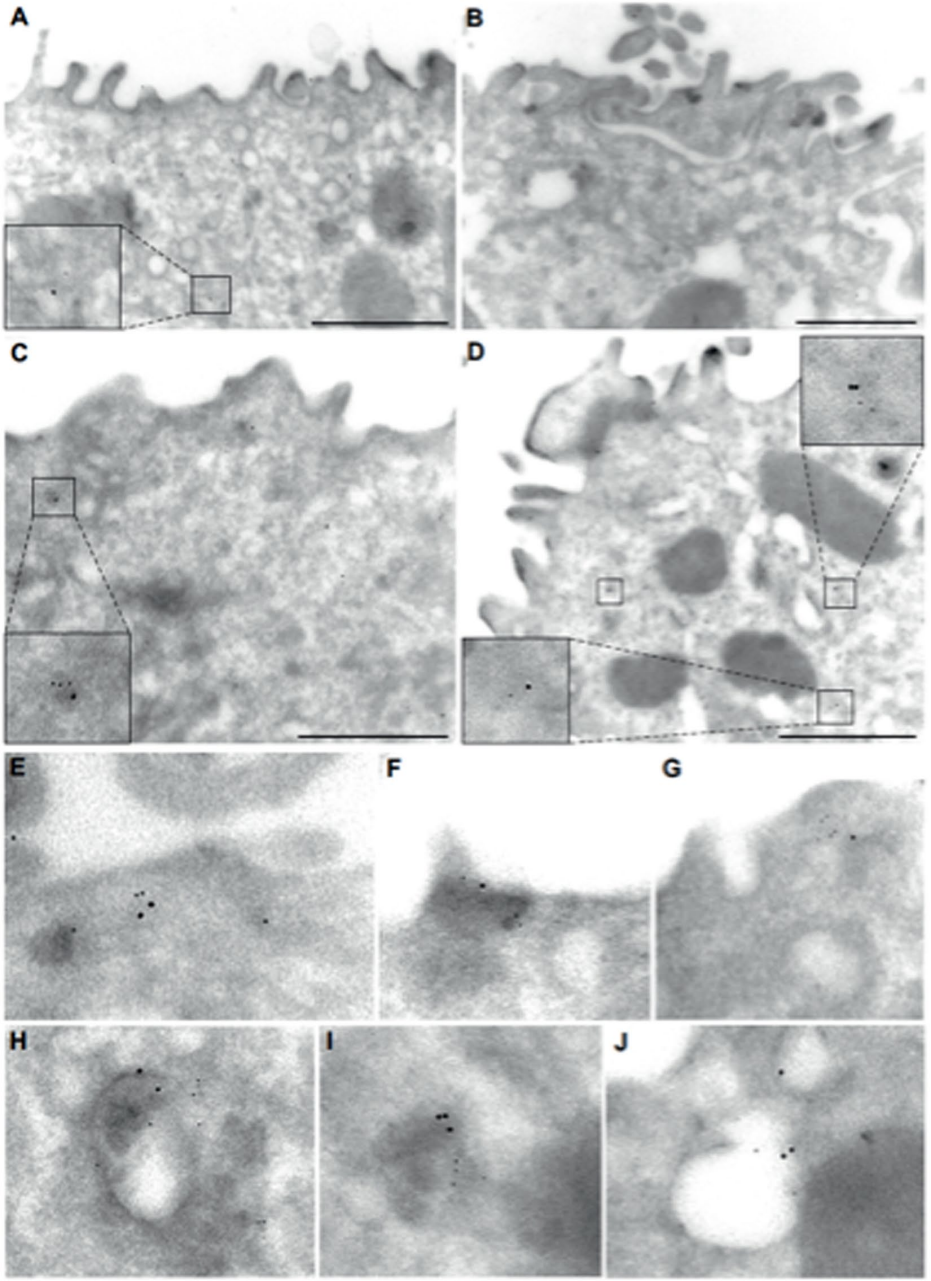
Electron microscopy detects close proximity of NCC and ENaC in murine kidney cortex after aldosterone treatment. Immunogold labeling of NCC (12 nm particles) and γENaC (6 nm particles) in mouse DCT2 of (**A**,**B**) vehicle (25% DMSO in saline) and (**C**–**J**) Aldo-treated kidney cortex. The inserts (**A**–**D**) show a magnified view of closely associated NCC and γENaC. Figures **E**–**J** are magnified views of increased NCC- γENaC intracellular colocalization, close to the cell surface (**E**–**G**) and in intracellular vesicles (**H**–**J**). Scale bar = 1 μm.

Given the importance of Aldo in regulating both NCC and ENaC, we hypothesized that Aldo would modulate their interactions in the DCT2. Therefore we treated mice on a high salt diet (4%) with Aldo or vehicle for 10 days and EM immunogold labeling was performed^12^. High salt diet was used to suppress systemic renin-angiotensin II- aldosterone (RAAS) levels. Apical images were obtained from the DCT2, the only portion of the nephron containing both NCC and ENaC. As demonstrated previously, under control conditions NCC and γENaC (NCC, 12 nm; γENaC, 6 nm) were occasionally found in close proximity on the apical surface of the DCT (Fig. 1A,B; inset). When mice were treated with Aldo, we observed an approximate 3 fold increase in NCC and ENaC colocalization (0.03 mean occurrences control *vs*. 0.08 mean occurrences in Aldo-treated) (Fig. 1C,D; inset and E–J). These qualitative data suggest that hormonal stimulation modulates NCC and ENaC interactions.

### Aldosterone treatment increases NCC-ENaC coimmunoprecipitation *in vitro* and *in vivo*

**Figure 2:**
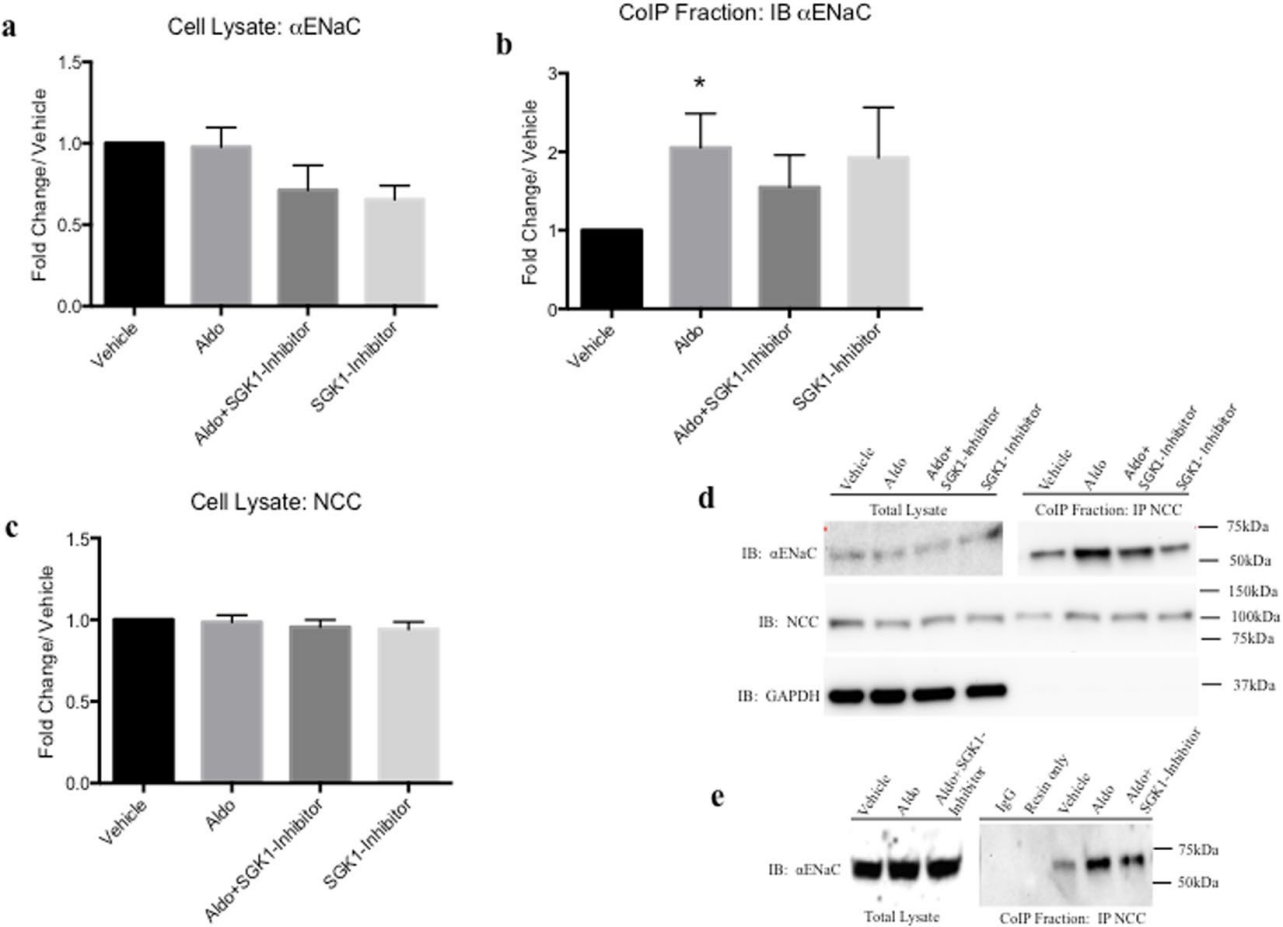
Coimmunoprecipitation demonstrated increased association of NCC and αENaC in mouse distal convoluted cells with Aldo stimulation. Total cell lysate of mDCT15 cells were immunoprecipitated (IP) with anti-NCC antibody and probed with αENaC antibodies in vehicle, Aldo-treated (100 nM, 48 hrs), Aldo + SGK1 inhibitor (GSK690394, 6 uM, 60 min) or with SGK1 inhibitor alone. (**a**) mDCT15 total lysate probed with αENaC. (**b**) mDCT15 cell lysate IP with NCC and probed with αENaC antibody (**c**) mDCT15 cell lysates probed with NCC following respective treatments (**d**) Representative blots for CoIP experiments. Blots were cut to assay for multiple antibodies. (**e**) No antibody lanes contain lysate with resin only. Separation in images shown with a space. Data represented as mean ± SEM, n = 5; Wilcoxin Signed-Rank, *p < 0.05.

Having shown that physiological levels of Aldo treatment increased interaction of NCC and ENaC using EM we examined whether Aldo alters their interaction by CoIP *in vivo* and *in vitro*. In Fig. 2, CoIP experiments were performed with whole cell lysates from mDCT15 cells treated with Aldo (100 nm, 48 hrs) or vehicle (EtOH). Previously, we have confirmed the specificity of our protocol and showed that Na/K-ATPase (α subunit) and GAPDH do not immunoprecipitate with NCC^12^. When comparing the fold changes in αENaC protein expression with Aldo treatment in total lysate, no significant changes were observed compared to vehicle treatment (0.98 ± 0.12 fold change/vehicle, n = 4) (Fig. 2a,d). A slight, yet insignificant decrease was found with Aldo + SGK1-inhibitor (0.71 ± 0.15 fold change/vehicle, n = 3) However, as shown in Fig. 2b and d, Aldo stimulation resulted in increased αENaC immunoprecipitation with NCC (2.05 ± 0.43 fold change/vehicle, p < 0.05, n = 4–5). Similarly, NCC total protein expression was unchanged with Aldo treatment (0.99 ± 0.04 fold change/vehicle, n = 3–4) or co-treatment with Aldo and the SGK1 inhibitor (0.97 ± 0.04 fold change/vehicle, n = 3–4). When we compared the αENaC and NCC present in the CoIP fractions, we observed an approximate 1.5 fold increase in αENaC present (1.55 ± 0.20 fold change/vehicle, n = 3). This suggests that the increased interactions observed between NCC and αENaC are not a simple function of increased total protein expression.

To examine the Aldo-dependent increases in NCC-αENaC interactions, we used an SGK1 inihbitor (GSK690394, 6 uM, 60 min). Following Aldo + SGK1 inhibition, we observed a trend for reduced NCC-αENaC interaction (2.05 ± 0.43 Aldo *vs*. 1.55 ± 0.41, Aldo + SGK1 inhibitor, Fig. 2). For all experiments, no signifcant changes were observed in GAPDH expression, nor were interactions observed with Co-IP resin only (no antibody, representative blot shown Fig. 2e). These data suggest a quantitative increase in NCC-αENaC interaction with Aldo stimulation. With SGK1 inhibition, there is a trend for a reduced interaction.

**Figure 3:**
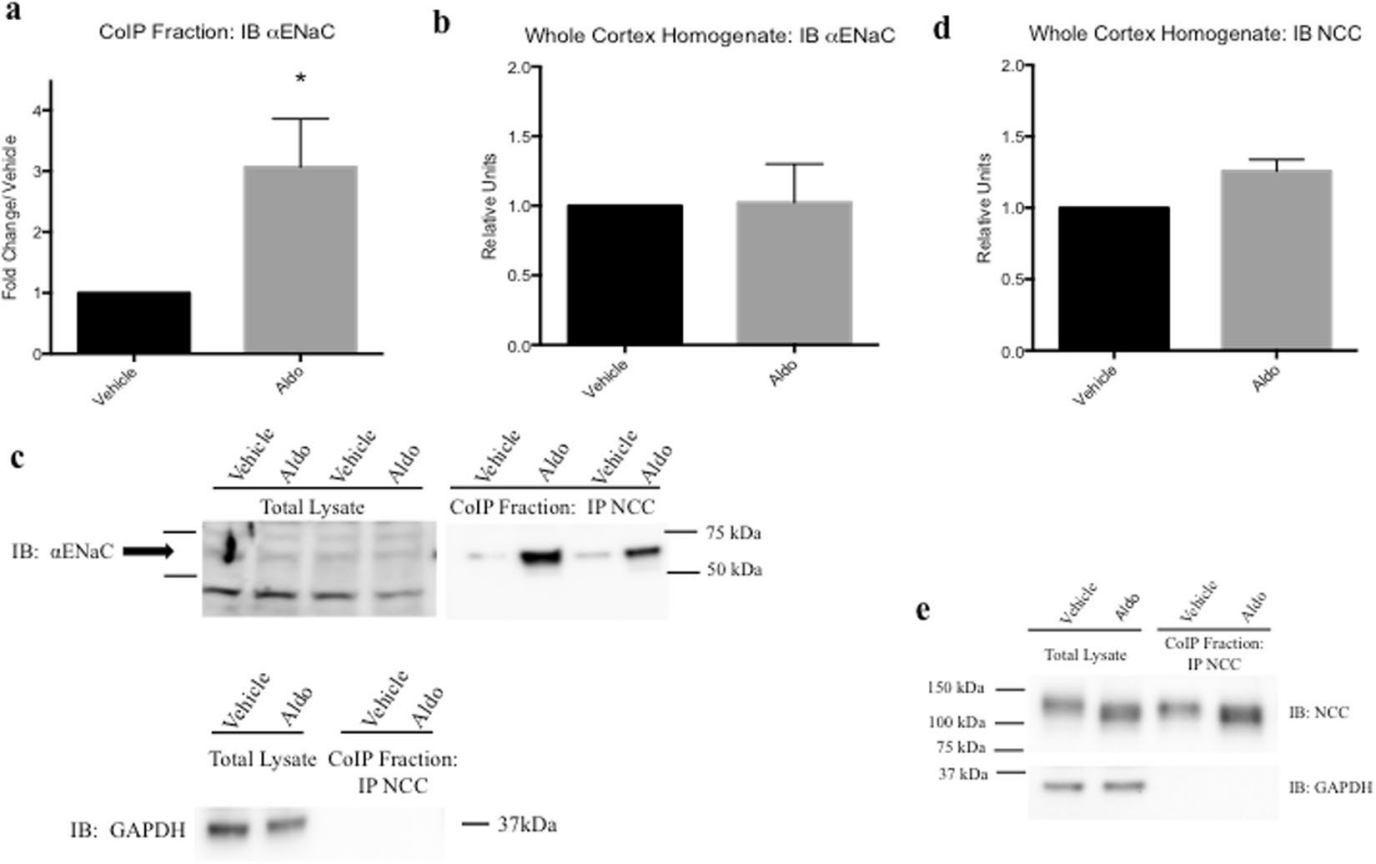
Coimmunoprecipitation demonstrated increased association of NCC and αENaC aldosterone-treated mice. (**a**) Mouse kidney cortex homogenates were used and CoIP performed; CoIP fraction probed with αENaC antibodies in vehicle (25% DMSO in saline) or Aldo-treated (4 μg) mice for 10 days, fed high salt (4%) chow. (**b**) Whole kidney cortex lysate probed with αENaC. (**c**) Representative blots for CoIP experiments. Blots were cut to assay for multiple antibodies. (**d**) Whole kidney cortex homogenate probed with NCC and (**e**) representative blots for NCC (lysate and and CoIP fraction) and loading control (GAPDH). Blots were cut to assay for multiple antibodies. Separation in images shown with a space. Data represented as mean ± SEM, n = 6; Wilcoxin Signed-Rank, *p < 0.05.

Although the mDCT15 cell line is the best available DCT2 culture model, we also explored this phenomenon *in vivo*. Mice were treated with Aldo via mini-osmotic pump (4 ug/day, 10 d) or vehicle (25% DMSO in saline) and were fed high salt chow (4%) to reduce systemic Aldo and AngII production. Following Aldo treatment, whole cortex homogenates were used for CoIP studies^12^. NCC and αENaC protein interactions were increased with Aldo treatment (3.07 ± 0.79 fold change/vehicle, *p < 0.05, n = 6, Fig. 3a,c). Furthermore, this increased interaction was not accompanied by an appreciable increase in αENaC expression in total cortex lysate (Fig. 3b,c). When looking at NCC expression in whole cortex homogenates, there was a trend for increased NCC total protein amounts, yet no significant increases were observed (Fig. 3d). These data confirm that the increased interactions observed between NCC and αENaC with Aldo treatment are not a function of increased total protein expression.

### NCC and αENaC subunit interactions are modulated via aldosterone-sensitive pathways

We have demonstrated that NCC and αENaC increase their biochemical interactions following Aldo treatment. To investigate whether the direct binding of NCC and ENaC was also regulated by aldosterone signaling pathways, we performed mammalian 2-hybrid assays using transiently transfected COS-7 cells, and a dual-luciferase reporter assay. In addition to baseline NCC-α, γ ENaC interactions, we assessed whether SGK1 and WNK4 affected their binding. COS-7 cells were transiently transfected with either: empty vectors (pBIND, pACT), positive (Myo-pACT + Id-pBIND) and/or NCC-pBIND, αENaC or γ-pACT, SGK1 or WNK4. This model of SGK1 overexpression was used to simulate increased Aldo-mediated MR activity since COS-7 cells have limited functional expression and/or activity of the MR.

**Figure 4:**
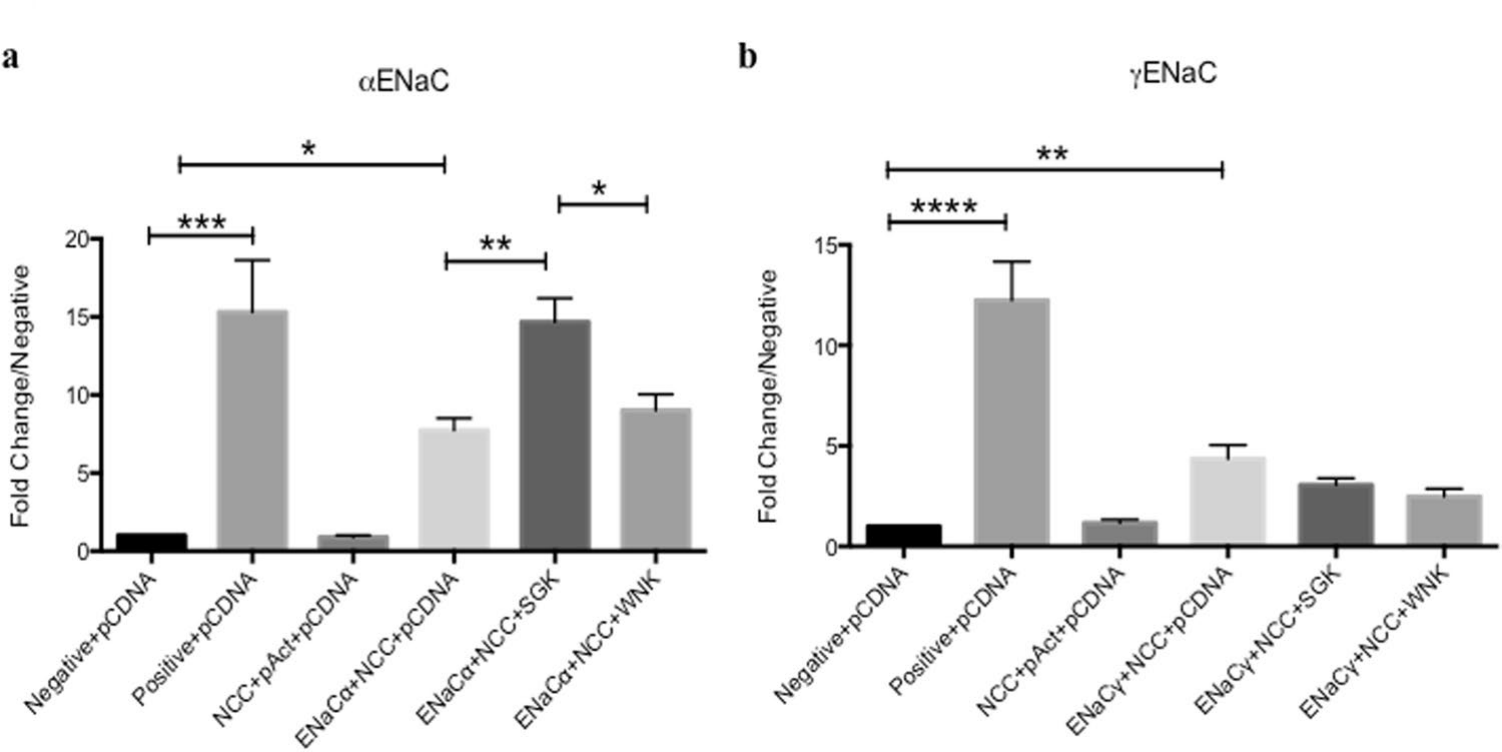
Mammalian two-hybrid demonstrates direct SGK1-mediated NCC and ENaC interaction. Mammalian two-hybrid assay performed on transiently transfected COS-7 cells with: empty vectors (pBIND, pACT), positive (Myo-pAct + Id-pBIND) and/or NCC-pBIND, αENaC (4A) or γ-pAct (4B), SGK1 or WNK4. Empty vector pCDNA was used to normalize for total amounts of DNA transfected. COS-7 cells lysed and values of Firefly and Renilla luciferase obtained. Firefly luciferase data were normalized to Renilla and all data expressed as fold change of negative control group (empty vector pBIND + pAct). Data represented as mean ± SEM, n = 7–12, at least 3 separate experiments: (**a**) ANOVA, *p < 0.05, **p < 0.01, ***p < 0.0001, (**b**) Kruskal-Wallis, **p < 0.01, ****p < 0.0001.

We observed a similar baseline interaction (Fig. 4a) between NCC and αENaC (7.74 ± 0.77 *vs*. negative, n = 7–12, ***p < 0.0001). With the additional transfection of the SGK1 vector, we saw a further increase in direct NCC-αENaC interaction with observed values (14.68 ± 1.58 *vs*. NCC-αENaC alone, n = 7–12, *p < 0.05) reaching values similar to positive control-transfected groups, supporting our previous data obtained using a different mammalian 2-hybrid system/reporter, and now confirming that Aldo-sensitive pathways increase NCC/ENaC interactions. When cells were co-transfected with WNK4, as a control for other proteins that regulate NCC, no increase in interaction was observed. This demonstrates the specificity of the SGK1-Aldo mediated pathways.

To investigate whether this process occurs with the γ subunit, similar experiments were performed. As shown in Fig. 4b, NCC and γENaC exhibit a consistent baseline interaction (4.33 ± 0.67 *vs*. negative, n = 8–12, **p < 0.01). In contrast, co-expression of either SGK1 or WNK4 produced no changes in NCC-γENaC interaction.

## Discussion

NCC and ENaC have similar regulatory pathways, frequently exhibiting converging mechanisms of regulation and their roles in maintaining Na^+^ balance cannot be understated. The overlap of NCC and ENaC expression occurs only along the second portion of the DCT, or DCT2. Understanding the regulation of these two transport proteins within the distal nephron is crucial in developing paradigms for distal electrolyte transport.

Both NCC and ENaC are regulated by the primary sodium conservation hormone Aldo, which is synthesized in the zona glomerulosa of the adrenal cortex and is an essential part of the RAAS system^13^. During times of hypovolemia, feedback mechanisms stimulate the production of angiotensin II (AngII) and Aldo. With a reduction in volume, increased Aldo production occurs, leading to activation of several important pathways. Given this regulation of both NCC and ENaC by Aldo, we hypothesized that Aldo would also modulate their interaction. To investigate this, we first determined if Aldo stimulation would lead to increased NCC-ENaC proximity for interaction (colocalization) in the DCT2 of murine cortex using EM (Fig. 1). We found a qualitative increase in colocalization with Aldo treatment, as compared to kidneys from vehicle-treated mice.

Recently, investigators have shown that the regulation of NCC by Aldo may occur only in DCT2. This is largely due to the presence of 11β-HSD2. This enzyme converts cortisol to inactive cortisone, conferring ‘Aldo-sensitivity’ to a particular cell^14^. Studies have shown that 11β-HSD2 is not expressed in the DCT1; however, Poulsen and colleagues demonstrate that 11β-HSD2 is clearly expressed in the DCT2, but show that the expression levels were not as high as the CNT or the CCD^15, 16^. It should be noted that the 11β-HSD2 expression level, under high Aldo conditions, is currently unknown in the DCT2. Together, these data suggest that Aldo-sensitivity may exist in the DCT2 where both NCC and ENaC are localized. In addition, this preferential Aldo effect of the DCT2 could also be because of non-genomic Aldo effects, or secondary to potassium (K^+^) concentrations. However, the reasons why K^+^ levels would affect the DCT2 *vs*. the DCT1 (where 11β-HSD2 is clearly not expressed) are unclear. Regardless of the mechanism, there is a clear differential affect of DCT1 *vs*. DCT2.

Others have suggested that DCT2/CNT localized ENaC is largely insensitive to Aldo; however in that manuscript, those authors were unable to completely distinguish between the DCT2 and CNT^17^. We think this is an important distinction, as we have previously demonstrated that ENaC properties are altered in the presence of NCC (HCTZ reduced ENaC P_O_)^12^, as would be the case in the DCT2 and not CNT. Thus, while ENaC regulation in the CNT appears insensitive to Aldo, we believe this effect has not been fully examined in DCT2. Here, we reveal an Aldo-dependent regulation of NCC and ENaC interaction within the DCT2. Therefore one such interpretation of these data are that increased NCC may lead to increased ENaC activity in a portion of the distal nephron where ENaC is not classically Aldo-sensitive.

To quantitatively determine if this increased interaction is independent of increased protein expression, we performed CoIP studies using mDCT15 cells, which natively express DCT2 signaling pathways and kidney cortex from sham and Aldo-treated mice. Aldo treatment significantly increased NCC and αENaC interaction in both mDCT15 cells as well as murine kidney cortex (Figs 2 and 3). Surprisingly, in our model using Aldo-treated mice fed a HS (4%) diet, no increase in αENaC expression (Fig. 3b–d) was observed in whole cortex homogenates. However, similar results were observed with adrenalectomized rats infused with Aldo (200 ug/day + HS, 4 days). In those studies, Wang and colleagues also found that αENaC protein expression was not increased^18^. Other investigators have also shown that Aldo treatment increases DCT2 NCC protein expression levels to that of the DCT1^15^. Here, our data also suggest that Aldo can increase NCC-αENaC interaction.

Aldo mediates intracellular signaling via activation of the intracellular mineralocorticoid receptor (MR)- a member of the nuclear receptor superfamily^19, 20^. Activation of the MR produces dimerization and nuclear translocation where it participates in the activation of a variety of genes which regulate the increase in Na^+^/K^+^ ATPase, ENaC and ROMK channels, including SGK1^21^. SGK1 is expressed ubiquitously, and is regulated by a variety of factors increasing Na^+^ reabsorption^22, 23^. Some studies have suggested that SGK1 can directly phosphorylate ENaC subunits, although this has not been conclusively determined^24^. However, it is known that SGK1 potently stimulates increased ENaC at the apical membrane, and possibly increases open probability (P_O_). ENaC transcription is likewise increased with SGK1 activation^25, 26^. Most importantly, ubiquitinylation by NEDD4–2 leads to internalization and degradation of ENaC and NCC via proteosomal degradation. Active SGK1 phosphorylation of NEDD4–2 reduces Nedd4–2 activity, thereby increasing NCC and ENaC membrane abundance^23, 27^. In our *in vitro* model, we used pharmacological inhibition of SGK1 before performing CoIP experiments. Interestingly, SGK inhibition exhibited a trend for reducing this interaction *in vitro*, although not significantly. However, in Fig. 4a, we co-transfected NCC and αENaC with SGK1 and performed M2H experiments. The expression of SGK1 dramatically increased direct NCC and αENaC interactions. We can only speculate that our *in vivo* experiments with Aldo + HS treatment increased SGK1 levels; if so, the M2H data support our CoIP findings where SGK1 inhibition showed a trend for reducing their interaction and *in vivo* where Aldo + HS increased interactions. The differences observed between the *in vitro* CoIP and M2H experiments may indicate the complicated regulation of the SGK1 pathway; however, strong SGK1 stimulation leads to increase NCC-αENaC interactions.

In order to determine if αENaC is the predominant binding partner, similar experiments were performed with NCC and γENaC (Fig. 4b). Surprisingly, only interactions were seen at baseline. There was no augmentation with SGK1 overexpression. These results suggest that increased interactions via Aldo may be mediated through the α subunit or that Aldo affects the binding of γENaC through a non-SGK1-mediated mechanism. Overall, these data show that Aldo increases NCC-ENaC interactions and reveal new mechanistic insight into distal Na^+^ handling. Given our new understanding of the molecular interactions that exist between NCC and ENaC, determining how they might be co-regulated is paramount.

Our laboratory has demonstrated that these two transport proteins not only exist in a macromolecular complex, but consistently interact^12^. Additionally, this novel and previously unsuspected occurence has raised other multiple important questions which need to be further investigated. In summary, we have explored the hypothesis that the NCC-αENaC interaction can be dynamically modulated via Aldo, and that this interaction is not static. We speculate that this study may reveal a reason why thiazide diuretics are so efficacious in hypertensive patients; thiazides are inhibiting the increased Aldo-mediated activation of NCC with ENaC in the DCT2. Previously we have shown that using a thiazide diuretic inhibits the activity of both NCC and ENaC, when they are associated. And here, we demonstrate that during conditions of increased Aldo, this interaction is increased. It is potentially during these circumstances, where there is increased ENaC interaction with NCC, thiazides would have a greater ability to inhibit both NCC and ENaC in the DCT2.

## Materials and Methods

### Materials

Chemicals were purchased from Sigma-Aldrich (St. Louis, MO), unless stated otherwise.

#### Cell Culture and Treatments

mDCT15 cells were plated on cell culture dishes and grown in medium containing a 50:50 mix of DMEM/F12 plus 5% heat-inactivated fetal bovine serum (FBS) and 1% penicillin/streptomycin/neomycin (P/S/N), at 37 °C. Experiments were conducted when the cells reached 90–95% confluence.

#### Standard Immunoblotting

mDCT15 cells were incubated as above. The cells were harvested, lysed using buffer containing protease inhibitor, and homogenized by sonication on ice. The cell lysates were centrifuged briefly and supernatant was collected. Proteins were transferred electrophoretically to PVDF membranes. After blocking with 3% BSA, the membranes were probed with corresponding primary antibodies; anti-NCC^28^ 1:4000–1:8000, Actin (Cell Signaling, 1:1000) overnight at 4 °C. All ENaC antibodies (1:200–1:1000 dilution) utilized for immunoblotting were from the laboratory of Dr. Douglas Eaton and have been validated^29–31^. The NCC antibody (1:4000–1:8000) was developed and validated in the laboratory of Robert Hoover^28^. The blots were washed in TBST (tris-buffered saline, tween 0.5%) and secondary antibodies were HRP-linked (Amersham, 1:5000). Supersignal West Pico was used for chemiluminescence (Thermo Scientific). Chemiluminescence was detected with G:Box (Gelbox) and analysis by Genetools software (Syngene, Frederick MD).

#### Animal Preparation and Experimentation

The Emory University Institutional Animal Care and Use Committee (IACUC) approved all animal protocols; experiments, methods and aseptic surgeries were performed according to those guidelines set by IACUC and all animal welfare regulations. Mice were kept in cages with autoclaved bedding and received free access to water and a standard diet (Diet 5001; Purina, 0.4% Na) or high salt diet (4%, Teklad Harland Laboratories, Indianapolis IN). Mice were implanted with miniosmotic pumps (Alzet, CA) containing aldosterone (4 μg/day, 25% DMSO, 10days, n = 6 mice/group). Sham mice were implanted with miniosmotic pumps containing vehicle solution; both groups were supplemented with high salt (4%) chow to reduce systemic Aldo production. Mice were euthanized using carbon dioxide, approved through Emory IACUC (IACUC protocol #DAR-2002607-012417BN). Kidneys were then harvested and the cortex dissected.

#### Electron Microscopy

Electron microscopy (EM) studies were performed using perfused kidneys from vehicle and Aldo-infused mice, all fed high salt (4%) chow. Kidneys were fixed by *in vivo* perfusion of 3% PFA/PBS, followed by post-fixation of kidney slices with 3%PFA/0.1% glutaraldehyde/PBS for 2 hours and cryoembedding. Ultrathin sections were blocked with serum-free protein block (Dako) followed by double staining with guinea pig anti-NCC antibody and rabbit anti-γENaC antibody (gifts of Johannes Loffing)^32^. Secondary antibodies were colloidal gold labeled anti-guinea pig (12 nm) and anti-rabbit (6 nm) both from Jackson laboratories. Images were captured with a Phillips CM-100 TEM.

To analyze approximate increases in NCC- γENaC colocaliztion, the DCT2 was identified from the location within the renal cortex via morphological appearance of the epithelium, and the coexistence of NCC and γENaC labeling. Using the open source software Image J (NIH), images of immunogold labeling of NCC (12 nm gold particles) and γENaC (6 nm gold particles) were quantified by the occurrence of NCC or ENaC particles at the plasma membrane and expressed as gold particles per plasma membrane length, and by the occurrence of NCC or γENaC particles intracellularly expressed as gold particles per cell area. This was performed in both control (15 sections counted) and Aldo-treated (27 sections counted) kidneys. The proximity of both proteins close enough for interaction (colocalization) was defined as distance between NCC and γENaC-gold particles <40 nm and expressed as occurrence per cell area. A distance of <40 nm was used, because of the biochemical approach using the linker technology where proximity of 2 proteins <40 nm is necessary to hybridize their primary antibodies used for detection, as desribed previously^33^.

#### Co-immunoprecipitation (CoIP)

mDCT15 cells or mouse kidney cortex (n = 6 mice/group) was lysed and BCA protein quantification performed. Total amounts of protein lysate (cells or whole cortex homogenates) used for the CoIP experiment were exactly the same for each sample (≈800 μg/experimental group per column) in the experiment. The resin beads were coupled to NCC, and then the samples were immunoprecipitated using all included buffers and reagents, according to the manufacturer’s instructions (Pierce Co-Immunoprecipitation Kit). Following, the total lysate/cell homogenates and corresponding eluted CoIP fractions were electrophoresed in equal quantities for each experiment (20 μg protein for each cell lysate and 20 μL for each CoIP fraction) and immobilized to PVDF membrane for immunoblotting with αENaCα, NCC or GAPDH. Antibodies for immunoprecipitation were the same as used for immunoblotting.

#### Preparation of Kidney Cortex

Mouse kidney cortex was homogenized in a glass tissue grinder in ice-cold RIPA buffer (1× protease inhibitors cocktail & 1× phosphatase inhibitor, ThermoScientific, Waltham, MA). After centrifugation (13,000 rpm, 20 min, 4 °C) protein concentrations were determined using BCA Protein Assay kit (ThermoScientific). The appropriate amount of each sample was diluted in a Tris-glycine/SDS sample buffer (125 mM Tris, pH 6.8, 4% SDS, 10% glycerol, 3% β-mercaptoethanol, 0.02% bromophenol blue) and heated at 70 °C for 15 min prior to loading for SDS-PAGE.

#### Mammalian Two-hybrid Assay

We utilized the Checkmate Mammalian Two-hybrid (M2H) assay kit (Clontech, Madison WI). A full-length clone for NCC was inserted into the DNA-binding domain (pBIND) plasmid vector, pBIND, to form a fusion protein with DNA-binding domain, GAL4 (NCC-pBIND). The pBIND vector also contains *Renilla reniformis* luciferase under the control of the SV40 promoter, to control for transfection efficiency. The pACT vector contains the herpes simplex virus VP16 activation domain upstream of a multiple cloning region, where full-length clones of each subunit of ENaC (α-, β- or γ-pACT) were inserted. Both vectors were sequenced to confirm sequence alignment before experimentation (GeneWiz, South Plainfield, NJ). Empty vectors (pBIND/pACT), not containing ENaC subunits, were used in negative control experiments. COS-7 cells were transiently transfected with these vectors, along with the pG5*luc* vector, which contains five GAL4 binding sites upstream of a minimal TATA box upstream of the firefly luciferase gene (*luc*+). In addition, cells were co-transfected with either With no-lysine kinase 4 (WNK4) or serum- and glucocorticoid-inducible kinase 1 (SGK1) overexpression vectors. To ensure total transfected DNA amounts were the same in each experiment, pCDNA was used to equalize transfected quantity. Positive control vectors containing myo and id (Myo-pACT, Id-pBIND) which are two proteins known to highly interact, were supplied by Clontech and used to control for assay. 48 hours after transfection cells were lysed and binding was assessed by measuring reporter gene expression (*firefly* luciferase) via Dual-Luciferase Reporter Assay System (Clontech, Madison WI), per manufacterer’s instructions.

#### Statistical Analysis

Western blot data from CoIP experiments were quantified and expressed as fold change over the vehicle-treated group. Statistical analysis was performed using the GraphPad software package (La Jolla, CA). Error bars represent SEM. Data were analyzed for statistical significance using a paired *t-test*, ANOVA (with Tukey’s posthoc analysis), Wilcoxin-Signed Rank or Mann-Whitney Rank Sum/Kruskal-Wallis (with Dunn’s posthoc analysis), where appropriate. A p-value of less than 0.05 was considered statistically significant.

## References

[1] Chobanian AV (2003). The Seventh Report of the Joint National Committee on Prevention, Detection, Evaluation, and Treatment of High Blood Pressure: The JNC 7 Report. JAMA.

[2] Simon DB (1996). Gitelman’s variant of Bartter’s syndrome, inherited hypokalaemic alkalosis, is caused by mutations in the thiazide-sensitive Na-Cl cotransporter. Nat Genet.

[3] Lifton RP, Gharavi AG, Geller DS (2001). Molecular mechanisms of human hypertension. Cell.

[4] Eladari D, Chambrey R, Picard N, Hadchouel J (2014). Electroneutral absorption of NaCl by the aldosterone-sensitive distal nephron: implication for normal electrolytes homeostasis and blood pressure regulation. Cell Mol Life Sci.

[5] Biner HL (2002). Human Cortical Distal Nephron: Distribution of Electrolyte and Water Transport Pathways. J Am Soc Nephrol.

[6] Loffing J (2001). Distribution of transcellular calcium and sodium transport pathways along mouse distal nephron. Am J Physiol Renal Physiol.

[7] Loffing J, Kaissling B (2003). Sodium and calcium transport pathways along the mammalian distal nephron: from rabbit to human. Am J Physiol Renal Physiol.

[8] Schmitt R (1999). Developmental expression of sodium entry pathways in rat nephron. American Journal of Physiology - Renal Physiology.

[9] Obermuller N (1995). Expression of the thiazide-sensitive Na-Cl cotransporter in rat and human kidney. Am J Physiol.

[10] Loffing J (2000). Differential subcellular localization of ENaC subunits in mouse kidney in response to high- and low-Na diets. American Journal of Physiology - Renal Physiology.

[11] Christensen BM (2010). Sodium and Potassium Balance Depends on αENaC Expression in Connecting Tubule. Journal of the American Society of Nephrology.

[12] Mistry AC (2016). The Sodium Chloride Cotransporter (NCC) and Epithelial Sodium Channel (ENaC) Associate. Biochem J.

[13] Root AW (2014). Disorders of aldosterone synthesis, secretion, and cellular function. Curr Opin Pediatr.

[14] Bostanjoglo M (1998). 11Beta-hydroxysteroid dehydrogenase, mineralocorticoid receptor, and thiazide-sensitive Na-Cl cotransporter expression by distal tubules. J Am Soc Nephrol.

[15] Poulsen, S. B. & Christensen, B. M. Long-term aldosterone administration increases renal Na+ -Cl- cotransporter abundance in late distal convoluted tubule. *Am J Physiol Renal Physiol* ajprenal 00352 02016, doi:10.1152/ajprenal.00352.2016 (2016). 10.1152/ajprenal.00352.2016 27733368

[16] Ackermann D (2010). *In vivo* nuclear translocation of mineralocorticoid and glucocorticoid receptors in rat kidney: differential effect of corticosteroids along the distal tubule. American Journal of Physiology - Renal Physiology.

[17] Nesterov V (2012). Aldosterone-dependent and -independent regulation of the epithelial sodium channel (ENaC) in mouse distal nephron. Am J Physiol Renal Physiol.

[18] Wang XY (2001). The renal thiazide-sensitive Na-Cl cotransporter as mediator of the aldosterone-escape phenomenon. J Clin Invest.

[19] Ferrario CM, Schiffrin EL (2015). Role of mineralocorticoid receptor antagonists in cardiovascular disease. Circ Res.

[20] Rautureau Y, Paradis P, Schiffrin EL (2011). Cross-talk between aldosterone and angiotensin signaling in vascular smooth muscle cells. Steroids.

[21] Viengchareun S (2007). The mineralocorticoid receptor: insights into its molecular and (patho)physiological biology. Nucl Recept Signal.

[22] Lang F, Artunc F, Vallon V (2009). The physiological impact of the serum and glucocorticoid-inducible kinase SGK1. Curr Opin Nephrol Hypertens.

[23] Lang F (2006). (Patho)physiological significance of the serum- and glucocorticoid-inducible kinase isoforms. Physiol Rev.

[24] Diakov A, Korbmacher C (2004). A novel pathway of epithelial sodium channel activation involves a serum- and glucocorticoid-inducible kinase consensus motif in the C terminus of the channel’s alpha-subunit. J Biol Chem.

[25] Kamynina E, Staub O (2002). Concerted action of ENaC, Nedd4-2, and Sgk1 in transepithelial Na(+) transport. Am J Physiol Renal Physiol.

[26] Alvarez de la Rosa D, Zhang P, Naray-Fejes-Toth A, Fejes-Toth G, Canessa CM (1999). The serum and glucocorticoid kinase sgk increases the abundance of epithelial sodium channels in the plasma membrane of Xenopus oocytes. J Biol Chem.

[27] Arroyo JP (2011). Nedd4-2 Modulates Renal Na+-Cl- Cotransporter via the Aldosterone-SGK1-Nedd4-2 Pathway. Journal of the American Society of Nephrology.

[28] Ko B (2012). A new model of the distal convoluted tubule. American Journal of Physiology - Renal Physiology.

[29] Greenlee MM (2013). Estradiol activates epithelial sodium channels in rat alveolar cells through the G protein-coupled estrogen receptor. American Journal of Physiology - Lung Cellular and Molecular Physiology.

[30] Bao H-F (2014). ENaC activity is increased in isolated, split-open cortical collecting ducts from protein kinase Cα knockout mice. American Journal of Physiology - Renal Physiology.

[31] Alli, A. A. *et al*. *Phosphatidylinositol phosphate-dependent regulation of Xenopus ENaC by MARCKS protein*. Vol. 303 (2012). 10.1152/ajprenal.00703.2011 PMC3468524 22791334

[32] Wagner, C. A. *et al*. *Mouse model of type II Bartter’s syndrome. II. Altered expression of renal sodium- and water-transporting proteins*. Vol. 294 (2008). 10.1152/ajprenal.00613.2007 18322017

[33] Venkatesan JK (2010). Nicotinamide adenine dinucleotide-dependent binding of the neuronal Ca2+ sensor protein GCAP2 to photoreceptor synaptic ribbons. J Neurosci.

